# Understanding How Synthetic Impurities Affect Glyphosate Solubility and Crystal Growth Using Free Energy Calculations and Molecular Dynamics Simulations

**DOI:** 10.1021/acs.jpcb.5c06978

**Published:** 2026-03-03

**Authors:** Alejandro Castro, Ignacio Sanchez-Burgos, Nuria H. Espejo, Adiran Garaizar, Giovanni Maria Maggioni, Jorge R. Espinosa

**Affiliations:** † Department of Physical Chemistry, 16734 Universidad Complutense de Madrid, Av. Complutense S/N, Madrid 28040, Spain; ‡ Yusuf Hamied Department of Chemistry, University of Cambridge, Lensfield Road, Cambridge CB2 1EW, U.K.; § Data Science, 1569 Bayer AG, Alfred-Nobel-Straße 50, Monheim Am Rhein 40789, Germany; ∥ Crop Protection Innovation, Bayer AG, Kaiser-Wilhelm-Allee 1, Leverkusen 51373, Germany; ⊥ Multidisciplinary Institute, Complutense University of Madrid, Paseo Juan XXIII, 1, Madrid 28040, Spain

## Abstract

Glyphosate, the most widely used herbicide worldwide, crystallizes through complex intermolecular interactions that are strongly influenced by synthesis-derived impurities. Understanding this process at the molecular scale is critical for optimizing production, ensuring product quality, and assessing the environmental impact. Here, we employ direct coexistence molecular dynamics simulations and free energy calculations to elucidate how glycine—a prevalent synthesis byproduct—modulates glyphosate solubility and crystal growth in aqueous solutions. Our simulations identify two major mechanisms by which glycine hinders crystallization. First, direct coexistence simulations show that glycine preferentially adsorbs at crystal surfaces, hindering glyphosate attachment and slowing growth. Second, free energy calculations demonstrate that glycine enhances glyphosate solubility, reducing the supersaturation driving force to incorporate into the crystal phase. Experimental measurements corroborate our predictions, confirming both enhanced solubility and reduced crystallization kinetics in the glycine-bearing systems. These findings establish that glycine—typically considered an inert impurity—actively disrupts glyphosate crystallization by promoting its dissolution. More broadly, this integrated computational–experimental approach highlights the power of molecular simulations to disentangle impurity effects, interfacial phenomena, and solution thermodynamics in crystallization, providing molecular-level insights for optimizing industrial protocols and predicting agrochemical behavior under relevant environmental conditions.

## Graphical abstract

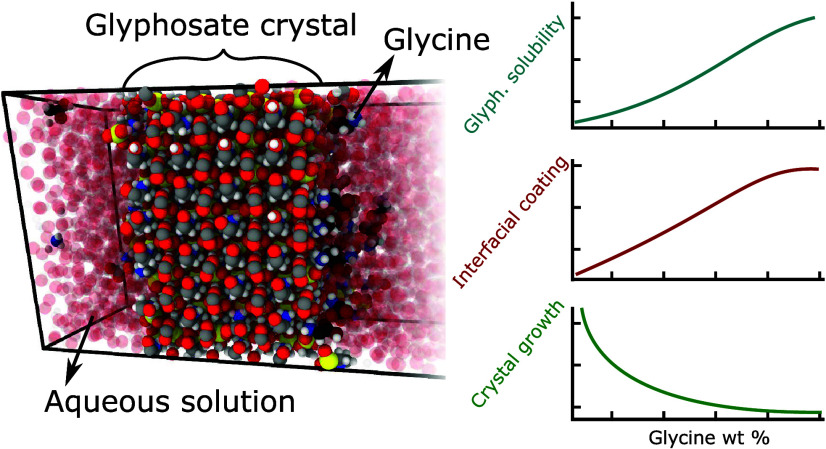

## 1 Introduction

Glyphosate (*N*-phosphonomethylglycine), the world’s most widely used herbicide,^1^ inhibits 5-enolpyruvylshikimate-3-phosphate synthase,^2^ an enzyme essential for biosynthesis of aromatic amino acids, including phenylalanine, tyrosine, and tryptophan, in a wide range of plants.^3^ The absence of this shikimate pathway in animals confers glyphosate’s selective toxicity toward plants.^4^ Structurally, glyphosate is a glycine analogue bearing a phosphonomethyl substituent at the nitrogen atom. Its molecular architecture incorporates carboxylic acid, amine, and phosphonic acid functionalities, conferring it an amphoteric character with multiple ionization states. Under environmental conditions (pH 6–8), glyphosate exists predominantly as a dianionic or trianionic species.^5^ Its high polarity and multiple ionizable groups drive strong interactions with both organic matter^6^ and soil minerals,^7^ influencing its environmental fate and strong bioavailability.

Crystallization represents a critical purification step in glyphosate synthesis, which determines product quality.^8^ While impurities do not directly affect its herbicidal activity, they are essential considerations for regulatory compliance and quality control.^9^ Impurities significantly influence crystallization kinetics through mechanisms that remain poorly characterized.^10^ This complexity arises from glyphosate’s pH-dependent zwitterionic structure,^11^ extensive hydrogen-bonding network,^12^ and polymorphic behavior under varying pH and pressure conditions.^13,14^ A molecular-level understanding of its solubility, nucleation rate, and crystal growth^15^ is therefore essential for optimizing crystallization protocols and elucidating impurity effects on crystal formation.^16^ In that sense, molecular dynamics (MD) simulations represent a powerful tool for obtaining relevant atomistic insights into these processes.

Here, we combine Direct Coexistence (DC) simulations with free energy calculations^17–20^ to investigate how glycine impurities control glyphosate crystallization. Free energy calculations are used to quantify the thermodynamic driving force for crystallization—i.e., relative supersaturation with respect to its solubility limit—estimating the relative glyphosate solvation free energy at different glycine concentrations. Moreover, through DC simulations, we model glyphosate crystals in equilibrium with aqueous solutions containing varying concentrations of glycine, providing insights into critical interfacial phenomena while yielding concentration-dependent solubility data. Together, these complementary MD approaches aim to reveal how glycine modulates glyphosate solubility from both thermodynamic and molecular perspectives. Furthermore, our experimental measurements confirm our computational predictions through a systematic evaluation of the saturation temperatures and induction times under controlled conditions. These experiments directly quantify glycine’s impact on solubility, nucleation kinetics, and growth rates, demonstrating an excellent agreement with computationally predicted trends. Our approach, integrating MD modeling and experimental characterization of glyphosate crystallization, establishes a relationship between impurity concentration and crystallization behavior in agrochemical production of glyphosate. Specifically, we elucidate the mechanisms through which glycine—a ubiquitous byproduct in glyphosate synthesis—impacts the solubility and disrupts the purification efficiency, providing actionable insights for further process optimization.

## 2 Materials and Methods

### 2.1 Models and Simulation Details

We perform DC simulations using the GROMACS 2023 MD simulation package.^21^ For the glyphosate–glycine aqueous solution systems, we use the OpenFF 2.0.0 force field^22–24^ using a total potential energy function (*U*_total_) that corresponds to the sum of the bonded and nonbonded interactions between the different atoms. For the nonbonded interactions, a 12–6 Lennard-Jones potential and Coulombic interactions are used. Water is modeled with the same type of potential interactions using the TIP3P water model included in the OpenFF 2.0.0 force field. Cross-interactions are handled via Lorentz–Berthelot mixing rules^25–27^ according to OpenFF 2.0.0. The LINCS algorithm^28^ is used to ensure bond constraints to any hydrogen atom. The molecular topologies used in these simulations were generated with the OpenFF toolkit.^23^ All simulations are performed at constant pressure (*p* = 1 bar) and temperature (*T* = 300 K). Direct Coexistence simulations are performed in the *NpT* ensemble using an anisotropic Parrinello–Rahman barostat^29^ with a relaxation time of 10 ps and a v-rescale thermostat^30^ with a relaxation time of 0.1 ps. The time step for the Leap-frog algorithm^31^ is 2 fs. For further details on the force field parameters, including the potential cutoffs and details on the Ewald summation algorithms (PME) for the long-range contribution of the electrostatic interactions, see the Supporting Information (SI) Section SI.

For the DC simulations, we place a bulk of crystal glyphosate pre-equilibrated on one side of the simulation box and water (or a glyphosate solution) on the remaining space of the simulation box, avoiding intermolecular overlapping between both phases.^32–36^ The exposed crystal orientation is either (010) or (001), which we specify in each corresponding section. We carry out two types of DC simulations: (i) including a perfect crystal of glyphosate in contact with a glyphosate solution; (ii) a glyphosate crystal with random vacancies introduced at the interface in contact with the solution. Once the system reaches equilibrium—normally after 100 ns—we compute density profiles along the long axis of the DC simulation box using the GROMACS tool *gmx density*.^21^ Then, the solubility (*m*_glyphosate_) is estimated as

1 $$m_{\mathrm{glyphosate}}=\frac{\rho^{\mathrm{glyphosate}}}{\rho^{H_{2}O}\times M_{\mathrm{glyphosate}}}$$

where ρ^glyphosate^ and $\rho^{H_{2}O}$ are the mass densities of glyphosate and water in the solution, respectively, and *M*_glyphosate_ represents the molar mass of glyphosate.

We additionally perform Free Energy Perturbation (FEP) MD calculations to estimate the solvation free energy of glyphosate in a series of different solutions with varying concentrations of glycine. This methodology provides accurate relative solubilities of small molecule active ingredients in a wide range of solvents and solutions, as recently shown in refs 17–19 . For these calculations, we use Schrödinger’s FEP+ implementation^37,38^ to visualize and setup atomistic simulations with 0.5%, 1%, and 2% glycine weight percentages and a single molecule of glyphosate. For these calculations, all interactions were parametrized using the OPLS4 force field,^39^ a well-established force field widely employed in free energy calculations.^17^ In these calculations, the solvation free energy is obtained from thermodynamic Hamiltonian integration^40^ through a combination of MD runs and postprocessing analysis using the Bennett Acceptance Ratio (BAR) method,^41^ which provides an efficient estimator of free energy differences from forward and reverse perturbations. We first equilibrate the different systems by running a ladder of different relaxation simulations, as described in Schrödinger’s solvation protocol.^18,42^ Temperature is kept constant throughout the simulations using a Nosé–Hoover thermostat^43^ with a relaxation time of 1 ps. The pressure is kept constant at *p* = 1 bar with an MTK barostat^44^ with a relaxation time of 2 ps. We sample 20 different replicas of each glycine weight percentage, allowing us to sample different local environments to accurately represent solution’s bulk properties. Further details regarding FEP+ calculations are provided in Section SII of the SI.

The link between the solvation free energy and solubility (described in eq 2) allows an indirect estimation of the relative solubility under different solution conditions:

2 $$\ln\left(\frac{m_{2}}{m_{1}}\right)=\beta \left(\Delta G_{1}^{\infty \;\mathrm{solv}}-\Delta G_{2}^{\infty \;\mathrm{solv}}\right)$$

where *m*_1_ and *m*_2_ represent the solubilities of a given solute in different solution conditions, β is the reciprocal of *k*_B_ *T* (where *T* is the temperature in Kelvin and *k*_B_ is the Boltzmann constant), and $\Delta G_{n}^{\infty \mathrm{solv}}$ is the solvation free energy of the compound in a given solvent *n* at infinite dilution.

### 2.2 Materials and Experimental Methods

Glyphosate was provided by the Bayer Crop Science department, with HPLC grade 95% purity. We measured the solubility and detection time using a Crystal16 instrument (Technobis Crystallization Systems, 2021). Here, the detection time is defined as the time elapsed between the discovery of the supersaturation point and the first detection of crystals. Solubilities were measured according to the following protocol: in a vial, selected amounts of glyphosate, glycine, phosphoric acid, and water were weighed and allowed to equilibrate at 293 K for 3 h. The temperature was then gradually increased at a rate of 1 K/min up to 333 K, and the point at which the solution became clear (the clear point) was interpreted as the saturation temperature. For the water-glyphosate system, the solubility measurements obtained via the clear point method were also confirmed by equilibrating a suspension at 304 K for 24 h and measuring the concentration of the liquid phase via HPLC.

We determine the detection time with a Crystal16, which is equipped with a laser to detect variations of light transmittance in the solution being monitored. This method is well-established experimentally to infer nucleation kinetics, and it has been extensively discussed in the literature. Further details on the method, assumptions, and limitations can be found in Kadam et al. and Maggioni et al.^45–47^ Specifically, each cycle consists of rapid heating from 293 to 333 K (30 min), a hold time at 333 K (60 min), rapid cooling back to 293 K (1 min), and a subsequent hold time at 293 K (360 min). Then, the detection time is evaluated from the moment 293 K was attained after rapid cooling. Because crystallization involves both nucleation and growth, which cannot be separately resolved with this technique, we adopt a simplified analysis. For each condition *j* with detection times *I*_*j*_, we define the growth time as

3 $$t_{\mathrm{growth},j}=\underset{i\in I_{j}}{\min}\left[t_{ji}\right]$$

i.e., the first observed detection time, assuming nucleation occurs at *t* = 0. The nucleation time for each experiment *i* is then calculated as

4 $$t_{n,ji}=t_{ji}-t_{\mathrm{growth},j}$$

assuming that growth time is constant under identical conditions. In this manner, nucleation and growth contributions can be distinguished, and their sums correspond to the overall detection time. Since our aim is to capture qualitative trends rather than exact nucleation kinetics, this approximation is appropriate for the present analysis.

## 3 Results and Discussion

### 3.1 Simulations Capture Crystal Density but Underestimate Glyphosate Solubility

Initially, we assess the force field’s ability to reproduce well-established properties of glyphosate, such as the crystal density, by preparing a 4 × 4 × 4 replication of the crystal unit cell^12^ (Figure 1a, left) at 300 K and 1 bar in the *NpT* ensemble. We then study how the bulk crystal density fluctuates throughout a simulation and compare it to the experimental data obtained by Wilson et al.,^13^ obtaining values in reasonable agreement (Figure 1a, right panel). The small overestimation of the simulated density (∼2%) is within the typical accuracy of a general-purpose fixed-charge force field,^48^ which has not been explicitly parametrized for a single molecule (e.g., glyphosate) crystal properties. Moreover, we determine that the crystal structure is well conserved throughout the MD simulation, preserving the crystal’s structural integrity and density over time.

**Figure 1:**
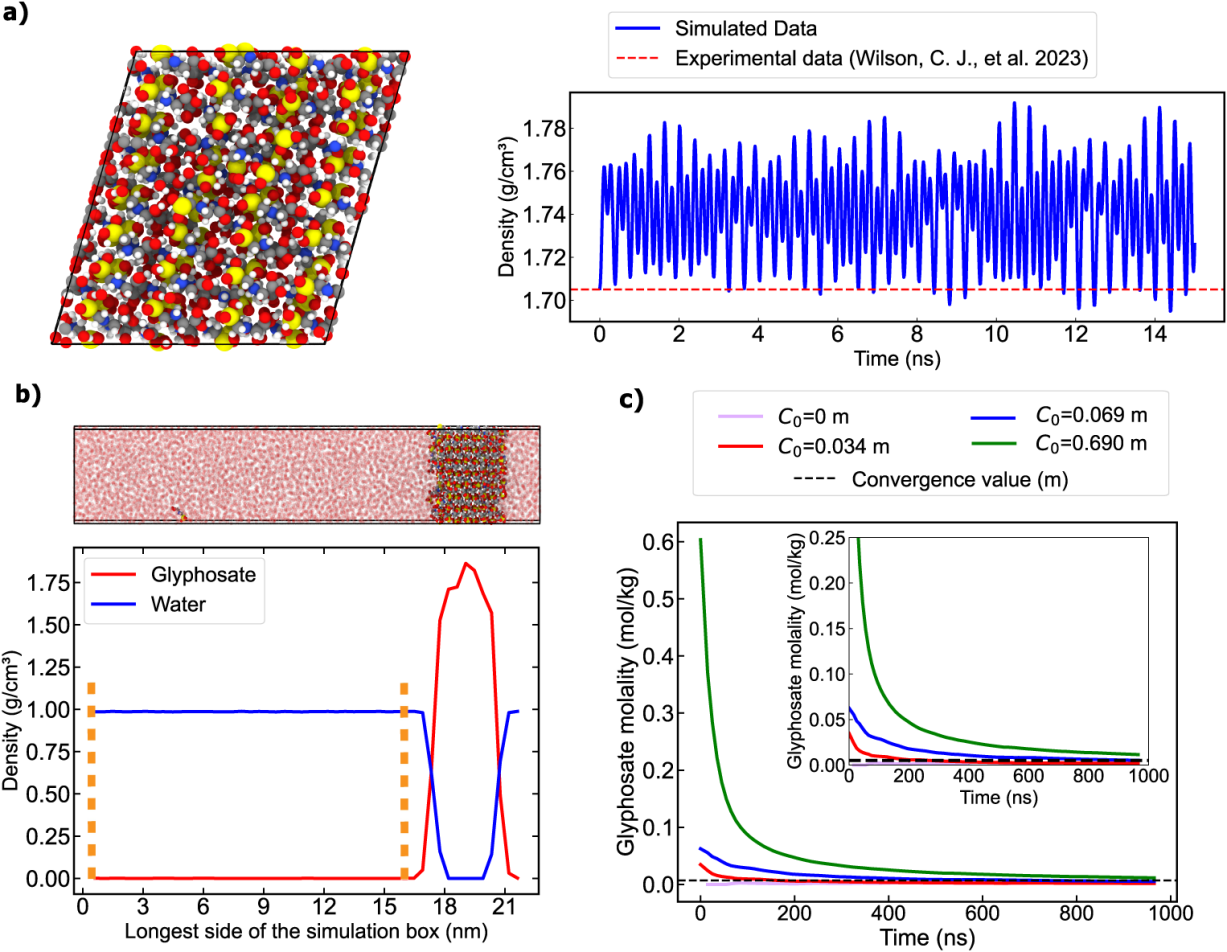
a) Left: Snapshot of the glyphosate bulk crystal structure^12^ from our simulations. Right: Time evolution of the crystal density from *NpT* simulations at 300 K compared to the experimentally determined value from ref 13 (dashed red horizontal line). b) Top: Representative snapshot from a DC simulation where water molecules have been rendered semitransparent to better visualize the glyphosate molecules in the bulk. Bottom: Density profile along the longest box direction of one of our systems (*C*_0_ = 0.069 m) without vacancies upon reaching equilibrium. The density profiles have been separated into different species, as indicated in the legend. The region that we define as the *bulk* solution is between the two dashed orange vertical lines. c) Time evolution of the glyphosate average concentration in the solution for different systems, each with a different initial glyphosate concentration in the bulk liquid phase. The values were obtained by calculating the converged average value of the concentration at all times < *n* time. The average value among all of the different trajectories (i.e., solubility limit, *m*) is indicated with a dashed horizontal line. The inset shows a zoomed-in view of the *Y*-axis.

Once we confirm that the crystal is accurately modeled and correctly maintains its crystal structure, we continue by preparing DC initial configurations exposing the (010) crystal orientation, where the crystal structure is in direct contact with different aqueous solutions, which contain varying concentrations of glyphosate in the solution. We corroborate that the initial glyphosate concentration in the solution does not affect the obtained solubility determination by performing multiple simulations with different initial concentrations of glyphosate. This is because in the *NpT* ensemble, the system spontaneously evolves toward equilibrium conditions, in which the crystal coexists with the solution at the saturation concentration.^49^ The prepared DC configurations contained 8000 water molecules and the necessary varying glyphosate replicas in the solution ranging from 0 to 0.690 mol/kg (further details of system sizes can be found in SI, Section SIX).

As discussed in Section 2, we use eq 1 to determine the solubility from the density profile along the long axis of the DC simulations, as illustrated in Figure 1b. Through orange dashed vertical lines, we bound the liquid phase from where we obtain the parameters that enter eq 1 to determine the glyphosate concentration in the solution (*m*). In Figure 1c, we show the time evolution of the glyphosate concentration (in molality units) within the solution. While all of the different simulations significantly differ in the initial glyphosate concentration, all trajectories converge to similar values within the uncertainty upon ∼600 ns. Importantly, we note that although stochasticity plays a role in the time needed for a given initial configuration to converge in the equilibrium state, such an effect becomes more pronounced when the initial solution concentration largely differs from the solubility limit^49^ as for the simulation starting from *C*_0_ = 0.690 m (green curve in Figure 1c).

Furthermore, we perform DC simulations, also exposing the (010) orientation, to examine how the presence of vacancies affects the kinetics to reach the equilibrium concentration of glyphosate in the solution. To build these systems, we randomly remove 10 glyphosate molecules from the outer crystal plane in contact with the solution, artificially creating defects on the surface. The same initial concentrations of the glyphosate solution depicted in Figure 1c are then used to determine the solubility from the density profiles once the systems reach equilibrium (i.e., the concentration of glyphosate in the solution shows no drift as a function of time). The obtained average results from these two types of simulations using multiple trajectories with different initial concentrations are summarized in Table 1 (further details on these calculations are shown in Figures S1 and S2 and Table S1). Based on these results, we do not find significant differences neither in the kinetics nor in the equilibrium value of the DC simulations in the presence vs absence of interfacial defects. Nevertheless, we note that in most of our defect-free simulations, the glyphosate molecules incorporated from the solution into the crystal do not adopt the correct bulk lattice structure.

**Table 1:** Comparison of the Experimental Density^13^ and Solubility^50^ Predictions, along with Computational Calculations (Using DC Simulations with a Crystal Phase in Absence versus Presence of Vacancies at the Interface)

	Crystal Density (g/cm^3^)	Solubility (mol/kg)
Experimental data	1.705	(62.1 ± 0.1) × 10^–3^
Simulated without vacancies	1.74 ± 0.04	(4 ± 3) × 10^–3^
Simualted with vacancies	-	(4 ± 3) × 10^–3^

We then examine how the glyphosate solubility obtained via DC simulations compares with experimentally reported values. Table 1 summarizes both the experimental results^13,50^ and our computational predictions for crystal density (in g/cm^3^) and solubility (reported in millimolality for consistency with the reference data). The OpenFF force field reproduces the crystalline density within 2% of the experimental value but significantly underestimates solubility by roughly an order of magnitude. This discrepancy is consistent with the general parametrization strategy of OpenFF, which does not explicitly target organic molecule solubilities.^13^ Indeed, even for simpler aqueous systems such as NaCl solutions, state-of-the-art force fields often underestimate solubilities, with deviations ranging from 10% up to an order of magnitude.^49,51–53^ Importantly, the convergence of simulations initiated at different glyphosate concentrations toward similar equilibrium solubility values reinforces that OpenFF systematically underestimates glyphosate solubility.

### 3.2 Calculation of Glyphosate Solubility Using DC Simulations Exposing Different Crystal Faces

As previously discussed, glyphosate crystallizes from an aqueous solution in a pH-dependent zwitterionic form, adopting a monoclinic unit cell. This structure belongs to the *P*2_1_/*c*1 space group in the Hermann–Mauguin notation. As shown in Figure 2a, crystal faces with different Miller indices exhibit distinct characteristics, such as the number of molecules directly exposed to the solvent and their interfacial free energy with the solution. In Section 3.1, we have focused on systems where the (010) crystal face of glyphosate was in contact with the aqueous phase. In this section, we investigate whether exposing a different crystal face, as illustrated in Figure 2a, may affect the determination of solubility. From a thermodynamic standpoint, the solubility limit of a crystalline solid in solution is independent of which crystal face is exposed. At equilibrium, the chemical potentials of the solid and dissolved species are equal, and solubility is therefore a bulk property of the coexisting phases rather than a surface-dependent quantity.^49,54^ Nevertheless, different faces present distinct interfacial free energies and growth rates,^55^ which can give rise to anisotropic growth and dissolution rates.^35,56^ In practice, such differences may influence kinetics, even though they should not alter the equilibrium solubility itself. Moreover, comparing the solubility values obtained from simulations using different crystal orientations provides an important internal consistency check. If calculations with distinct faces converge to the same solubility, this rules out the possibility that finite-size effects or kinetic trapping bias the results since it is highly unlikely that two independent orientations would be affected by the same artifact in an identical manner. Thus, studying multiple orientations not only probes potential kinetic effects but also strengthens confidence in the robustness of solubility determination.

**Figure 2:**
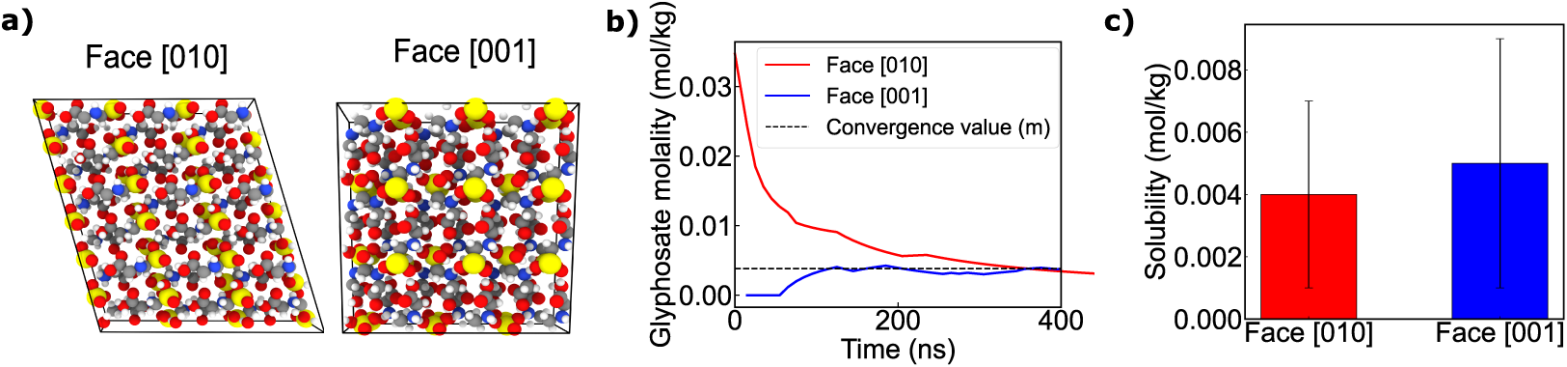
a) Snapshots of the different crystallographic planes studied in a 4 × 4 × 4 unit cell. b) Convergence of the molality of two independent DC simulations with different crystal orientations and different initial glyphosate concentrations as a function of time. The values were obtained by calculating the arithmetic accumulated average value of the solubility at all times < *n* time. The average values of both trajectories are depicted by a horizontal dashed line. c) Bar plot of the obtained solubility of the two different crystallographic faces. The whisker represents the uncertainty bounds.

To investigate potential kinetic trapping and finite-size effects in our systems, we consider an additional crystal orientation (001) and construct two new systems for DC simulations. In both cases, the number of water molecules was fixed at 8000, and the required number of glyphosate molecules in the solution was added to achieve initial concentrations of 0 and 0.69 m. As shown in Figure 2b, systems differing in both the exposed crystal plane and the initial glyphosate concentration converge to the same solubility value, indicated by the dashed line. Statistical analysis of the average solubilities for both orientations (Figure 2c) shows that the results are equivalent within the associated uncertainties. In summary, the crystal face in contact with the aqueous solution does not affect the solubility of glyphosate within the uncertainty, consistent with previous observations in other systems using DC simulations.^49^ This agreement between orientations indicates that our simulations are not kinetically trapped and that finite-size effects are negligible.

### 3.3 Glycine Impurities Increase Glyphosate Solubility

Real systems are more complex than just water and glyphosate, as they often contain glycine, a ubiquitous impurity introduced during glyphosate synthesis. At the pH where glyphosate crystallizes, glycine exists mainly in two forms: as a zwitterionic species and a negatively charged species, in a 1:4 ratio.^57^ To examine the effect of this impurity, we constructed four systems with varying glycine concentrations while maintaining a fixed number of water and glyphosate molecules in the solution. In addition, we consider both crystal orientations previously described in Sections 3.1 and 3.2. As before, we analyzed the DC simulations and obtained the density profiles of the new systems, shown in Figure 3a. Glycine is dynamically adsorbed onto the crystal surface through a continuous adsorption–desorption process, as evidenced by the two maxima highlighted in the inset of Figure 3a. This behavior is observed for both glycine forms (zwitterionic and negatively charged; see Figure S4). The snapshot in Figure 3a further reveals that, instead of aggregating into a solid-like structure on the glyphosate crystal, glycine forms a partial coating layer at the interface, characteristic of transient adsorption. We further characterize this behavior by measuring the surface concentration Γ = *N*_Glycine_/*A* for the (010) and (001) planes at two glycine concentrations (0.5 and 2 wt %). Our surface concentration measurements through DC simulations are summarized in Table 2. Based on our calculations, for a given concentration, the different exposed crystal planes exhibit similar adsorption capacities, as the values of Γ agree within the associated uncertainties. Nevertheless, higher glycine concentration leads to a greater surface concentration, a behavior that is known to further hinder crystal growth in similar systems.^58,59^ This prediction will be later confirmed in results from Section 3.4, where we experimentally determine the crystal growth rate as a function of the different glycine concentrations.

**Table 2:** Surface Concentration of Glycine (Γ) for Two Different Crystal Orientations of Glyphosate Measured at Two Glycine Concentrations Using DC Simulations

	Γ (molecules/nm^2^)	
Plane	0.5% wt Glycine	2% wt Glycine
(010)	0.11(10)	0.36(10)
(001)	0.18(10)	0.43(10)

**Figure 3:**
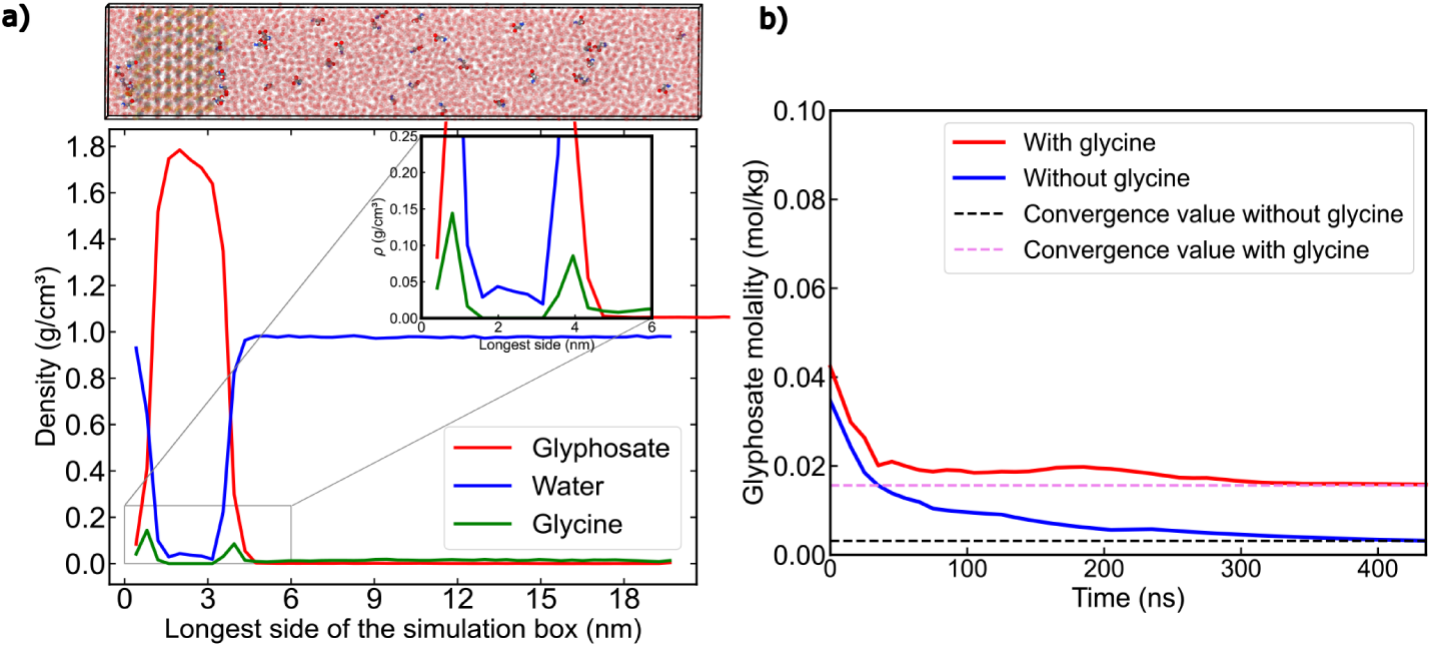
a) Top: Snapshot from a DC simulation where water and glyphosate molecules have been rendered semitransparent to better visualize the glycine molecules in the bulk and at the crystal surface. Bottom: Density profile of a DC simulation along the longest box direction, separated into its different components, as indicated in the legend. The inset presents a zoomed-in insight of the density profile showing the glycine density peaks at the crystal surface. b) Glyphosate concentration vs time for two different systems, one containing 2 wt % glycine (shown in red) and another in the absence of glycine (shown in blue). The values were obtained by calculating the accumulated average value of the glyphosate concentration at times < *n* time. The convergence value (solubility limit, *m*) is indicated with a dashed horizontal line (shown in violet and black in the presence vs absence of glycine, respectively).

In Figure 3b, we display the time evolution of glyphosate concentration converging to its solubility value in the presence (2 wt % glycine) and absence of glycine. The presence of glycine increases the solubility by approximately 1 order of magnitude. Importantly, this enhancement in solubility cannot be explained solely by the transient surface coating observed in Figure 3a hindering glyphosate attachment into the surface. We hypothesize that glycine adsorption may result from its concentration exceeding the solubility limit, leading to heterogeneous nucleation at the glyphosate–water interface. However, a detailed investigation of glycine solubility is beyond the scope of this study.

As commented in Section 3.2, solubility is determined by the equality of the chemical potentials of the crystal and dissolved molecules in the solution.^54^ The presence of glycine at the interface does not alter the chemical potential of glyphosate in either phase and, therefore, should not directly affect the solubility. Instead, glycine adhesion at the interface is expected to influence only the dynamic properties of the crystal, such as the growth and exchange rates. To further investigate the origin of the solubility change, we examined how glycine affects the solvation free energy of glyphosate in water. Solvation free energy is defined as the change in the Gibbs free energy when a solute is transferred from a vacuum to a given solvent/solution at a constant temperature and pressure. It quantifies the favorability of solute–solvent interactions. We calculate glyphosate solvation free energies using the FEP+ method described in Section 2.1. The systems spanned glycine concentrations from 0 to 2 wt %, and each contained 15,000 water molecules, one glyphosate molecule, and the corresponding number of glycine molecules required to reach the target concentration. The results, shown in Figure 4a, reveal a clear trend: the presence of glycine decreases the solvation free energy of glyphosate.

**Figure 4:**
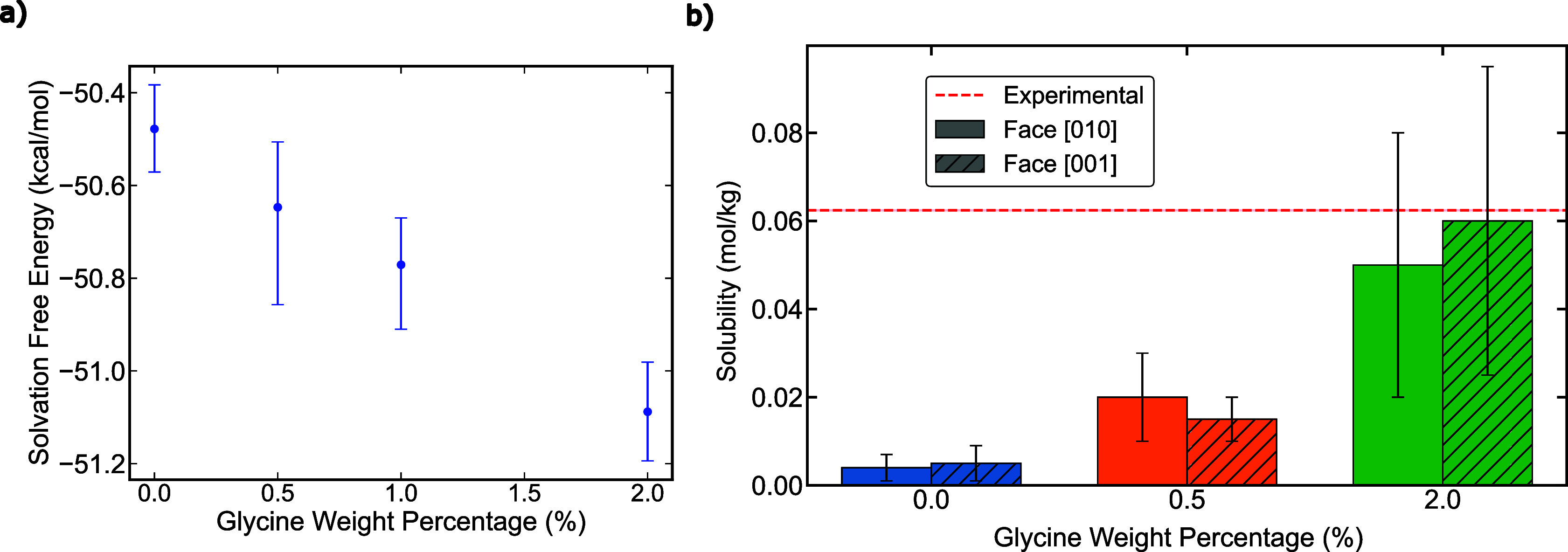
a) Solvation free energy as a function of glycine weight percentage from free energy calculations. We observe that increasing the glycine content leads to a further decrease in solvation free energy. b) Bar plot of the obtained solubility in the different studied systems with different glycine concentrations. The horizontal dashed line depicts the experimental solubility value reported in the absence of impurities.^50^

Therefore, the presence of glycine makes it more favorable for glyphosate to remain in solution compared with the impurity-free system. Moreover, this analysis reveals an inverse correlation between glycine concentration and solvation free energy: as the glycine concentration increases, the solvation free energy decreases. This result explains the increase in solubility observed in Figure 3b when glycine is added. In this case, the presence of impurities directly alters the chemical potential of glyphosate in solution. The change in solvation free energy is therefore linked to the solubility of the system, as shown in eq 2. This mechanism is also corroborated in Figure 4b, where we compare the solubility obtained with increasing glycine concentrations. We observe that the presence of glycine monotonically increases the glyphosate solubility by approximately an order of magnitude within the studied concentration range. Moreover, we also observe that, as expected, the solubility is not affected by the crystal orientation exposed to the solution, as already established in Section 3.2. Additionally, from our DC simulations, we computed the radial distribution functions between the different heavy atoms of glyphosate and glycine in order to determine which specific interactions are responsible for their intermolecular association that drives the solvation of glyphosate and adsorption of glycine to the crystal. These distributions are shown in Figure S7 and reveal that electrostatic interactions are crucial, being that between glyphosate’s oxygen and glycine’s nitrogen the most relevant one increasing both the solvation free energy (in absolute value) and the solubility as glycine concentration increases (see SM Figure S7).

Further comparison of our simulations with experimental data suggests that glycine, frequently present in glyphosate samples,^8^ may significantly influence solubility measurements. Using eq 2 and the 2 wt % glycine data, we estimate the solvation free energy corresponding to the experimentally reported solubility (62.4 ± 0.1 mmol/kg). Assuming a linear correlation between glycine concentration and solvation free energy (Figure 4a), we predict an effective glycine content of 2.7 ± 0.5 wt % in the experimental system, assuming that the OpenFF would predict the correct solubility for glyphosate. Although the actual glycine content of the experimental samples was not reported,^50^ this estimation provides a plausible explanation for the observed discrepancy in solubility—beyond force field deficiencies—and further strengthens the value of our computational framework for characterizing and predicting glyphosate phase behavior.

In summary, our results show that glycine hinders glyphosate crystallization through two distinct mechanisms. First, glycine dynamically adsorbs at the crystal–solution interface, coating the surface and potentially altering growth and exchange dynamics.^35,60,61^ Second, glycine lowers the solvation free energy of glyphosate, thereby making dissolution thermodynamically more favorable. Overall, glycine promotes glyphosate solubility in water by primarily modifying the solution thermodynamics.

### 3.4 Experimental Validation of the Simulation Predictions

To validate our simulation predictions, we conduct targeted experiments addressing two main objectives: (i) to confirm the enhancement of glyphosate solubility in the presence of glycine and (ii) to assess whether crystal formation kinetics are also slowed down, based on our hypothesis that glycine adsorbed at the crystal surface can interfere with crystal growth (Figure 3). For this purpose, the clear point temperature (equivalent to the saturation point) is determined in triplicate at constant glyphosate molality while varying the glycine concentration, following the procedure described in Section 2.2. Figure 5a shows the variation in the solubility temperature as a function of glycine concentration. The results indicate that glycine lowers the solubility temperature of glyphosate, thereby enhancing its solubility. Consequently, at a constant temperature, a higher glyphosate concentration is required to reach saturation, in full agreement with our simulation predictions in Figure 3.

**Figure 5:**
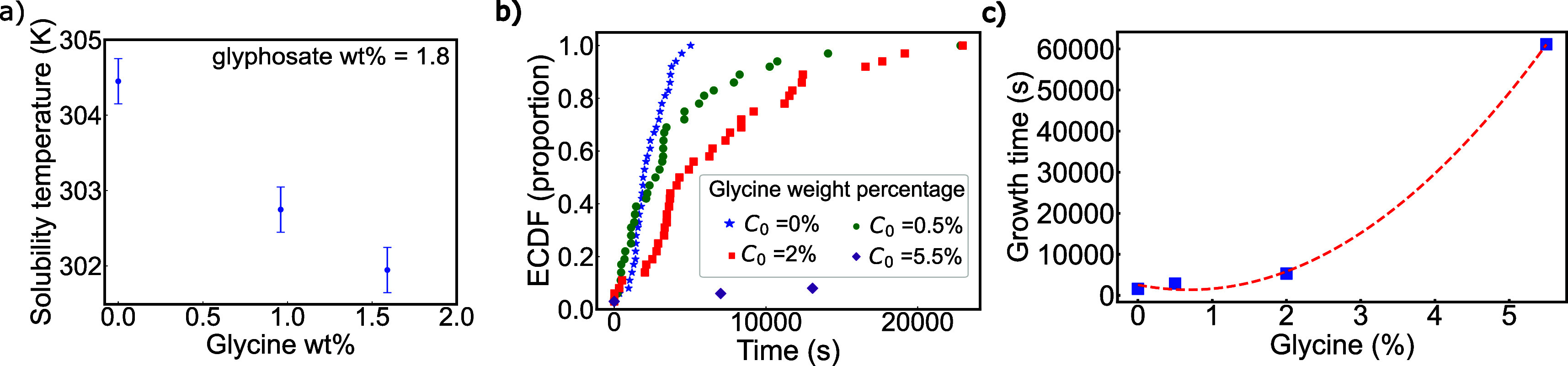
a) Solubility temperatures (measured as clear points) as a function of glycine percentage in weight. Experiments are performed at a constant concentration of glyphosate in acidic water for increasing concentrations of glycine. Our data refer to the mean values of triplicates, with the error bars indicating the associated standard deviation. b) Empirical cumulative distribution functions (ECDFs) of crystallization times obtained from the measured detection times at constant glyphosate supersaturation (*S* = 2) and varying concentrations of glycine, as indicated in the legend. The experiments are performed at a constant temperature, *T* = 293 K. c) Growth times obtained from the detection time analysis against the glycine weight percentage in the solution. A second-order fit, shown as a dashed red line, is included as a guide for the eye. The experimental uncertainty of these measurements is of the order of the symbol size.

Furthermore, to investigate the effect of glycine on the kinetics of glyphosate crystallization, we employ an indirect measurement approach based on detection times (see Section 2.2 for further details on the determination of this quantity). Regardless of the specific mechanism inhibited by glycine—primary nucleation or crystal growth—we hypothesize that the detection time distribution would shift toward longer times and may also broaden. To test this, we prepared systems with varying glycine concentrations and adjusted the corresponding glyphosate concentration to maintain a constant supersaturation of *S* = 2 at 293 K. The detection time is composed of two contributions: the nucleation time *t*_*N*_ and the growth time *t*_*G*_. The nucleation time corresponds to the interval between reaching the saturation point and the formation of the first nucleus, whereas the growth time is the interval between the nucleus formation and the detection of the crystals. While *t*_*N*_ is an intrinsic, hardly attainable property of the system, *t*_*G*_ depends on the monitoring technique employed. Further methodological details can be found in Kadam et al. and Maggioni et al.^45–47^ By applying the detection time analysis described in Section 2, we evaluate the distributions of nucleation and growth times from the overall detection time distributions. Figure 5B shows the empirical cumulative distribution functions (ECDFs) of nucleation events at three different glycine concentrations. The nucleation time distributions broadened, and their medians shifted toward larger values, indicating that nucleation became slower (i.e., less likely to occur within a fixed time), consistent with a reduced nucleation rate. Moreover, Figure 5C also presents the calculated growth times as a function of glycine concentration. Here, we observe that the growth time increases with glycine concentration, suggesting that the transformation of nuclei into observable crystals is also slower. Since the growth time primarily reflects the growth rate, these results indicate that the crystal growth rate decreases as the glycine concentration increases.

Taken together, our experimental observations show remarkable consistency with the results of our molecular dynamics simulations. This agreement is significant, as it not only validates the predictive capability of our simulation framework but also provides a molecular-level explanation for the observed macroscopic phenomena. Specifically, our simulations confirm that glycine plays a dual mechanistic role in modulating glyphosate crystallization. On the one hand, glycine alters the thermodynamic properties of the solution, enhancing solubility and thereby reducing the driving force for nucleation, as reflected in the broadening and temporal shift of the nucleation time distributions. On the other hand, the simulations reveal that glycine molecules preferentially colocalize at the surface of nascent glyphosate crystals. This surface association offers a plausible mechanistic explanation for the experimentally observed increase in growth time as the adsorbed glycine molecules are likely to hinder the incorporation of glyphosate molecules into the growing crystal lattice. The outcome is a reduction in the effective growth rate, consistent with the experimental trends. Thus, the synergy between the experiments and simulations enables us to propose a comprehensive mechanistic picture. The combined effects highlight the multifaceted role of impurities in crystallization processes and emphasize the importance of integrating molecular-level simulations with experimental validation. Beyond confirming the robustness of our findings, this approach provides a powerful framework for the rational design of crystallization modifiers in complex multicomponent systems.

## 4 Conclusions

In this work, we have investigated the molecular mechanisms by which glycine—a ubiquitous synthesis impurity—modulates the thermodynamics and kinetics of glyphosate crystallization in aqueous solution. By combining Direct Coexistence molecular dynamics simulations, free energy calculations, and experimental detection times of crystal growth, we show that glycine acts not as an inert byproduct but as an active crystallization inhibitor operating through dual pathways. Direct coexistence simulations reveal dynamic glycine adsorption at crystal–solution interfaces, forming transient partial layers that contribute to blocking glyphosate incorporation sites and hinder growth attachment. In parallel, free energy calculations demonstrate that glycine decreases glyphosate’s solvation free energy, thereby enhancing equilibrium solubility—as also demonstrated through Direct Coexistence simulations—and reducing supersaturation, the fundamental thermodynamic driving force for nucleation and growth. Together, these synergistic mechanisms establish glycine as both a kinetic inhibitor and a thermodynamic modulator of glyphosate crystallization.

Our simulations further show that glyphosate solubility is invariant across different crystallographic faces and insensitive to surface defects, confirming that glycine’s effects originate from bulk solution thermodynamics rather than interfacial artifacts. Experimental measurements quantitatively validate these predictions: increasing the glycine concentration systematically enhances solubility while hindering both nucleation and growth rates. This convergence between computation and experiment provides robust mechanistic validation. Overall, this work demonstrates the power of molecular simulations to disentangle the complex interplay between interfacial phenomena and solution thermodynamics in impurity-mediated crystallization. The mechanistic insights presented here—particularly the dual kinetic–thermodynamic inhibition pathway—offer molecular-level design principles for optimizing industrial crystallization processes and predicting impurity effects in pharmaceutical and agrochemical systems. More broadly, our framework establishes a quantitative methodology for understanding how synthetic byproducts influence crystallization in complex aqueous environments.

## Supplementary Material



## Data Availability

The data that support the findings of this study are available within the article and its Supporting Information. Topology files, MDP files, and some initial configurations are available in this GitHub link for the repository (https://github.com/Alex-castro-quim/Glyphosate_crystallization).

## Notes

The Supporting Information of this manuscript contains: The Supporting Information is available free of charge at https://pubs.acs.org/doi/10.1021/acs.jpcb.5c06978.

Detailed methods description (Sections SI–III); solubility calculations at different temperatures (Section SIV); simulations in the presence of glycine (Section SV); FEP+ calculation details (Section SVI); error calculation details (Section SVII); radial distribution functions (Section SVIII) (PDF)
