# Supplementary material for: Understanding How Synthetic Impurities Affect Glyphosate Solubility and Crystal Growth Using Free Energy Calculations and Molecular Dynamics Simulations
Source: J Phys Chem B. 2026 Mar 3;130(11):3217–26. doi: 10.1021/acs.jpcb.5c06978 (PMC13007038; doi:10.1021/acs.jpcb.5c06978)
Supplement: Supplementary file 1 [file jp5c06978_si_001.pdf]

Supplementary Material for *Understanding How Synthetic Impurities Affect Glyphosate Solubility and Crystal Growth Using Free Energy Calculations and Molecular Dynamics Simulations*

Alejandro Castro

Department of Physical Chemistry, Universidad Complutense de Madrid, Av. Complutense s/n, Madrid 28040, Spain

Ignacio Sanchez-Burgos

Yusuf Hamied Department of Chemistry, University of Cambridge, Lensfield Road, Cambridge CB2 1EW, UK

Nuria H. Espejo

Department of Physical Chemistry, Universidad Complutense de Madrid, Av. Complutense s/n, Madrid 28040, Spain and Data Science, Bayer AG, Alfred-Nobel-Straße 50, 40789 Monheim am Rhein, Germany

Adiran Garaizar*

Data Science, Bayer AG, Alfred-Nobel-Straße 50, 40789 Monheim am Rhein, Germany

Giovanni Maria Maggioni†

Crop Protection Innovation - Bayer AG, Kaiser-Wilhelm-Allee 1, 51373 Leverkusen, Germany

Jorge R. Espinosa‡

Department of Physical Chemistry, Universidad Complutense de Madrid, Av. Complutense s/n, Madrid 28040, Spain

Yusuf Hamied Department of Chemistry, University of Cambridge, Lensfield Road, Cambridge CB2 1EW, UK and

Multidisciplinary Institute, Complutense University of Madrid,
Paseo Juan XXIII, 1, Madrid 28040, Spain

(Dated: February 17, 2026)

* adiran.garaizarsuarez@bayer.com
† john.maggioni@bayer.com
‡ jorgerene@ucm.es

SI. THE OPENFF MODEL

We use the force field created by OpenFF toolkit[1, 2]. This initiative uses the Sage 2.0.0 force field format, defining molecular mechanic force fields using standard classical mechanic energy terms with a special parameter assignment system, a SMIRKS-based chemical perception of atom types. SMIRKS is a chemical substructure query language closely related to SMILES, instead of assigning atom types, OpenFF uses SMIRKS patterns to directly match chemical environments, so each parameter in the force field us attached to a SMIRKS pattern, not a defined atom type.[3] This effectively reduces the number of parameters needed and makes force fields eased to extend and maintain. The potential energy is computed as the sum of pairwise bonded (U_{bonded}) and non-bonded ($U_{\text{non-bonded}}$) interactions as:

$$U_{\text{total}} = U_{\text{bonded}} + U_{\text{non-bonded}} = U_{\text{bond}} + U_{\text{angle}} + U_{\text{torsion}} + U_{\text{vdW}} + U_{\text{electrostatics}}, \tag{S1}$$

where the bonded interactions are defined by the harmonic potential, the angle potential and the torsion potential, shown in

$$U_{\text{bond}} = \sum_{\text{bonds}} k_{\text{bond}}(r - r_0)^2, \tag{S2}$$

where r is the bond length, r_0 is the equilibrium bond length, and the spring constant k_{bond}. The sum runs over all paired atoms.

$$U_{\text{angle}} = \sum_{\text{angles}} k_\theta(\theta - \theta_0)^2, \tag{S3}$$

where θ is the bond length, θ_0 is the equilibrium bond length, and the spring constant k_θ. The sum runs over all angles formed by three consecutive linked atoms.

$$U_{\text{torsion}} = \sum_{\text{torsions}} \sum_{n} \frac{1}{2} V_n \left[1 + \cos\big(n\phi - \gamma\big)\right], \tag{S4}$$

where ϕ is the dihedral angle, V_n is the torsion barrier height, n is the periodicity and γ is the phase angle. The sum runs over all dihedral planes.

Non-bonded interactions consist of the sum of the hydrophobic interaction and electrostatic interaction. The hydrophobic interaction is given by the Lenard–Jones 12-6 form

potential. This potential is defined as

$$U_{\mathrm{vdW}}(r_{i,j}) = \sum_{i<j} 4\varepsilon_{ij}\left[\left(\frac{\sigma_{ij}}{r_{ij}}\right)^{12} - \left(\frac{\sigma_{ij}}{r_{ij}}\right)^{6}\right], \tag{S5}$$

where r_{ij} is the distance between atoms i and j, ε_{ij} is the depth of the potential well and σ_{ij} is the distance at which potential is zero.

Here σ_{ij} is the pair-of-beads diameter defined from the individual diameter (σ_i and σ_j) assuming the Lorentz-Berthelot mixing rules (i.e., $\sigma_{ij} = (\sigma_i + \sigma_j)/2$). The interaction parameter ε_{ij} is defined for each specific interaction thanks to Lorentz–Berthelot as $\varepsilon_{ij} = \sqrt{\varepsilon_i \varepsilon_j}$.

The electrostatic interactions are described by the Coulomb's law. This potential is defined as

$$U_{\mathrm{electrostatic}} = \sum_{i<j} \frac{q_i q_j}{4\pi\varepsilon_0 r_{ij}}, \tag{S6}$$

where q_i, q_j are the partial atomic charges and ε_0 is the vacuum permittivity. Cutoff for this interaction is 9 Å. Long-range electrostatic interactions were treated using the particle mesh Ewald (PME) method.

All simulations with this force field were carried out using the molecular dynamics Gromacs_mpi 2023.[4]

Topology files, mdp files and some initial configurations, where all parameters required to describe the intermolecular interactions, are available in this GitHub link for the repository.

SII. FEP+ AND OPLS4

For FEP+ (Free Energy Perturbations) calculus with OPLS4[5]. We created systems composed of 15000 solvent molecules, 1 solute molecule and the adequate quantity of glycine impurities for the different concentrations. 20 different seeds of the system were built to increase sampling and gather convergence statistics using the “tangled chain” option from Schrödinger Materials Studio.

For the FEP calculations, we use the default set up which creates 12 λ windows. λ is a coupling parameter that modifies the interaction energy of the system. A λ window is the simulation performed at a specific intermediate value of λ, representing a partially transformed state between the initial and final systems. At $\lambda = 0$, the system corresponds

to the initial non-solvated state, while at $\lambda = 1$ it corresponds to the final solvated state. In this case we modify the Van der Waals and Columbic contributions, meaning that at $\lambda = 0$ the interatomic interactions between solute and solvent are switched off, and at $\lambda = 1$, the interactions are fully switched on, and intermediate values represent scaled interactions. In the isobaric-isothermal ensemble (NpT) the difference in Gibbs free energy between two systems is given by equation S7[6].

$$G(N, p, T, \lambda = 1) = G(N, p, T, \lambda = 0) + \int_{\lambda=0}^{\lambda=1} \left\langle \frac{\partial U(\lambda)}{\partial \lambda} \right\rangle_{N,p,T,\lambda} d\lambda. \tag{S7}$$

where G is the Gibbs free energy, and U is the total energy.

To relax the system, we run a ladder of different equilibration simulations, as recommended by Schrödinger, in the solvation protocol[7]:

1. NVT Brownian Dynamics for 0.1 ns at 10.0 K and a timestep of 1 fs
2. Molecular Dynamics 0.024 ns/NVT/300.0 K/1 fs
3. Molecular Dynamics 0.24 ns/NVT/700.0 K/1 fs
4. Molecular Dynamics 0.024 ns/NPT/1.01325 bar/300.0 K/1 fs
5. Molecular Dynamics 0.24 ns/NPT/1.01325 bar/300.0 K/2 fs
6. Molecular Dynamics 20 ns/NPT/1.01325 bar/300.0 K/2 fs
7. Molecular Dynamics 10 ns/NPT/1.01325 bar/300.0 K/2 fs

Temperature is kept constant using a Nosé-Hoover chain thermostat[8] with a relaxation time of 1 ps and the pressure is kept constant with an MTK barostat[9] with a relaxation time of 2 ps. Equations of motion are integrated using RESPA[10], and short-range columbic interactions are cut at 0.9 nm.

Free energies are estimated with the BAR (Bennett Acceptance Ratio) method, using the fep_analysis script from Schrödinger tool set[7], which performs Hamiltonian integration calculations and yields the solvation free energy. The BAR method estimates free energy differences by combining forward and reverse energy samples between adjacent λ windows, providing a low-variance calculation of the total free energy change.

SIII. CALCULATION OF SOLUBILITIES VIA DIRECT COEXISTENCE SIMULATIONS

The determination of solubilities was performed using the Direct Coexistence (DC) method. The crystal is placed in a square prismatic elongated box where an aqueous solution of 8000 water molecules is put in contact with the liquid phase, where the molecule whose solubility we aim to calculate is disolved. The long side of the box is perpendicular to the interfaces.

Molecular Dynamic simulations were performed using Gromacs 2023 simulation package[4]. All runs were performed at 1 bar constant pressure and 300K constant temperature. DC simulations were performed in the NpT ensemble using an anisotropic Parrinello-Rahman barostat[11] with a relaxation time of 10 ps and a V-rescale thermostat[12] with a relaxation time of 0.1 ps. The time step for the Leap-frog algorithm[13] was 2 fs.

Through the study of density profiles obtained with the analysis tool *gmx density* we can identify different regions: the glyphosate crystal, the interface between the aqueous solution and the crystal and the aqueous solution bulk. The bulk of the aqueous solution is the region from which we obtain the partial densities to calculate the solubility through equation S8.

$$m_{glyphosate} = \frac{\rho_y^{glyphosate}}{\rho_y^{H_2O} \cdot M_{glyphosate}}, \tag{S8}$$

The density profiles for all studied systems are shown in figures S1 and S2

The results from applying equation S8 to the density profiles are summarized in table S1

SIV. OTHER INTERESTING SYSTEMS

Additionally we studied how different factors could affect glyphosate solubility. First, we study the effect of a 20 K increment in our simulations. The goal of doing this simulations is to check wether the system is kinetically arrested. In Fig S3.a we show the density profile for 320 K, from which we obtained the solubility results using S8. The comparison at 300 K ($4 \pm 3\, mmol/kg$) and 320K ($7 \pm 2\, mmol/kg$) yields interesting results. Despite showing the same solubility within the statistical error, we can see in Fig S3.b that a higher temperature promotes the existence of glyphosate in the solution bulk. We can also conclude that a 20 K increase is insufficient to produce statistically significant differences in our system, although

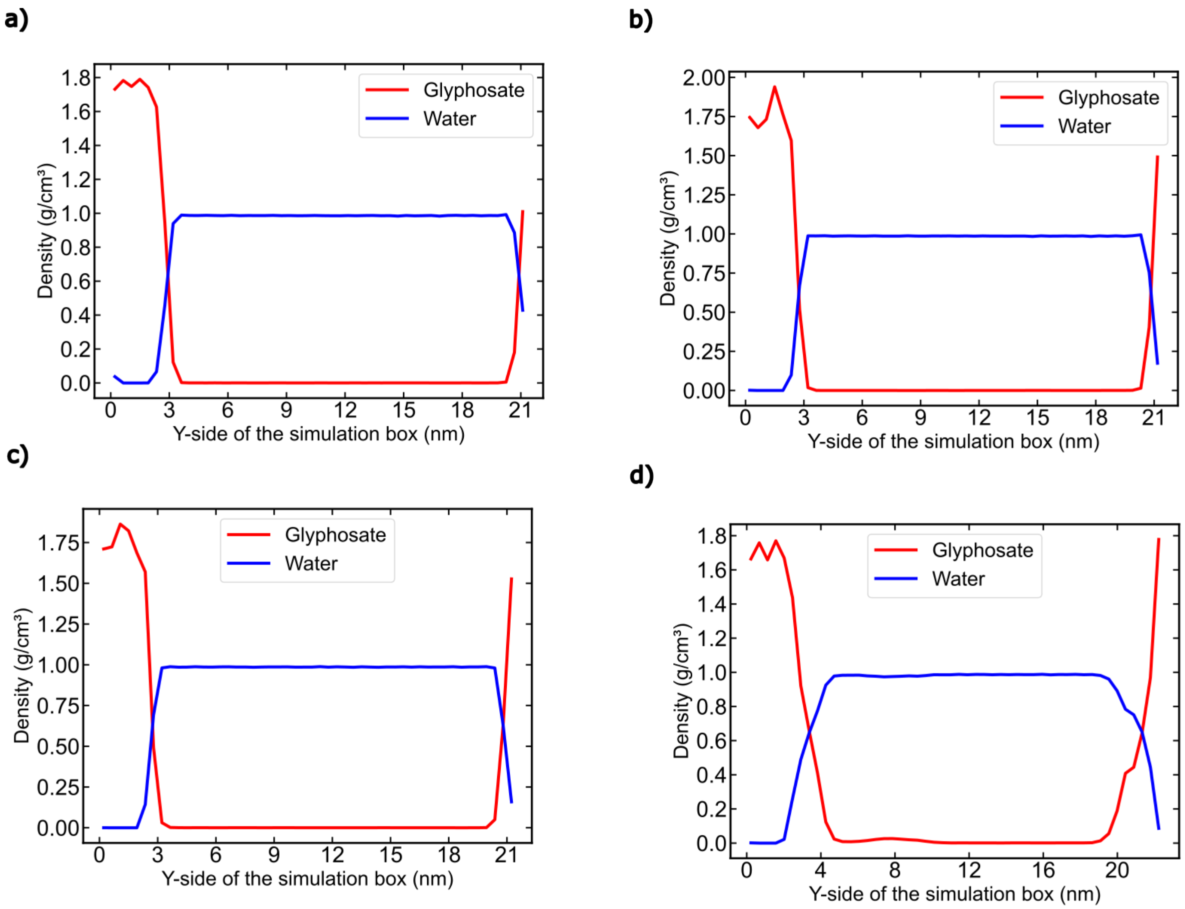

FIG. S1: All the non vacancy density profiles. a) $C_0 = 0\,mol/kg$ b) $C_0 = 0.034\,mol/kg$ c) $C_0 = 0.069\,mol/kg$ d) $C_0 = 0.69\,mol/kg$

it is enough to suggest a possible trend.

Moreover, we performed DC simulations for a system with a smaller cross section, implying a smaller interface area. Here we might find finite size effects to be relevant. Our simulations confirm this fact, as we find that there is no glyphosate molecule liberated from the crystal to the bulk of the aqueous solution, opposed to what we observe in Fig. S1.

SV. GLYCINE INSERTION

Glycine, the ubiquitous impurity present in glyphosate synthesis, must be taken into account in our simulations. As such, we need to study glycine at the experimental conditions used in glyphosate synthesis. At pH = 1.9, glycine shows two predominant forms, charged and zwitterionic[14], therefore, we took both species into account as separate entities for

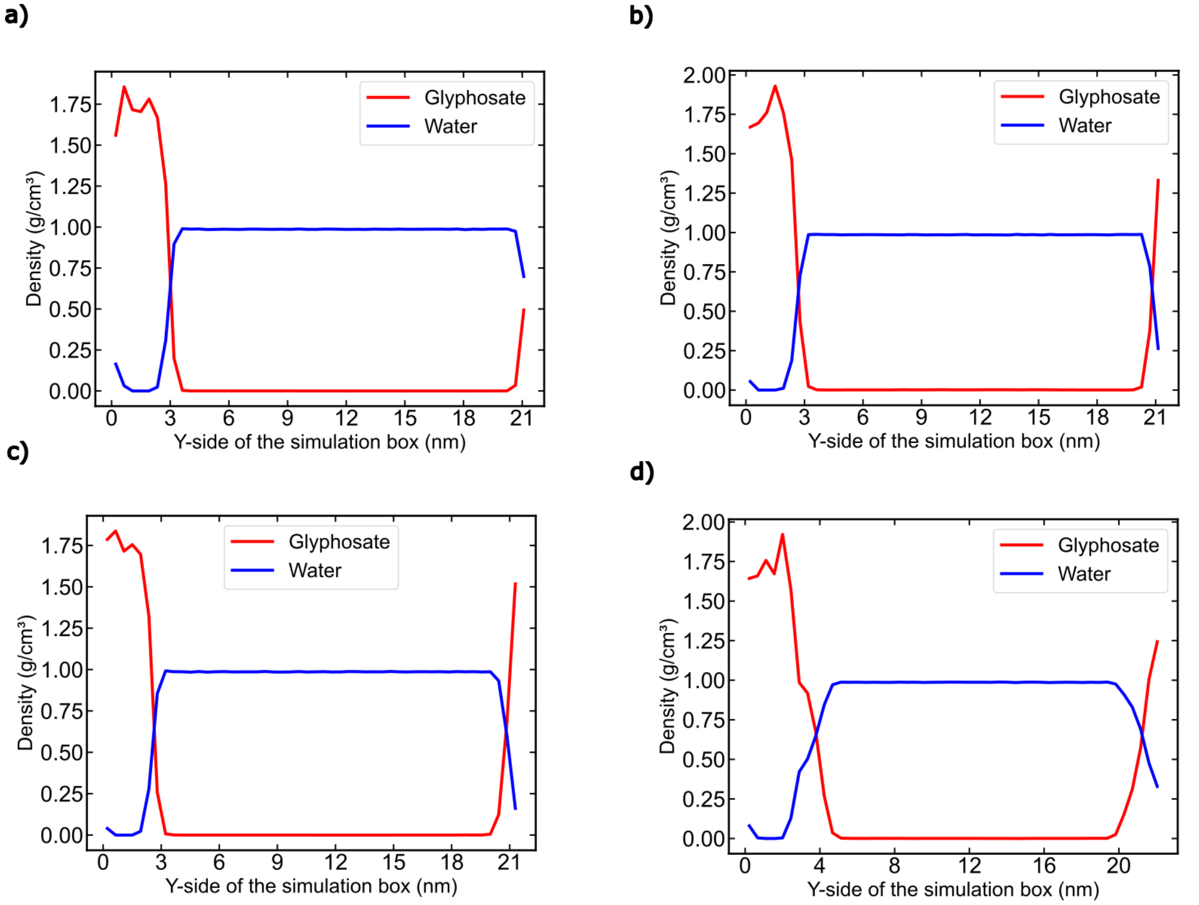

FIG. S2: All the vacancy density profiles. a) $C_0 = 0\,mol/kg$ b) $C_0 = 0.034\,mol/kg$ c) $C_0 = 0.069\,mol/kg$ d) $C_0 = 0.69\,mol/kg$

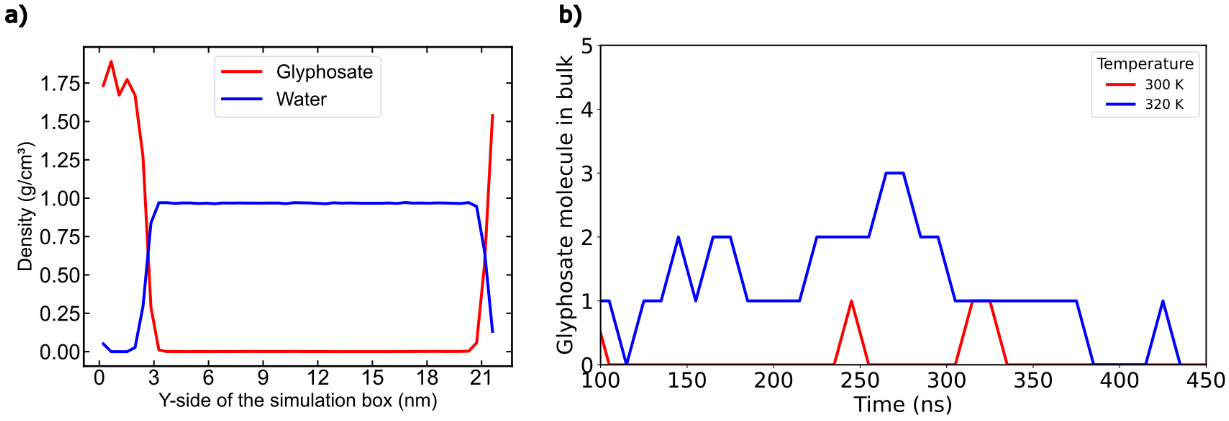

FIG. S3: Higher temperature experiments a) Density profile of a 320 K simulation. b) Comparison of the number of glyphosate molecules in bulk as a function of time for simulations conducted at 320 K and 300 K.

Simulation details	m_{gly} (mM)
0 glyphosate molecules added in a non-vacancy crystal	2.4 ± 0.5
5 glyphosate molecules added in a non-vacancy crystal	3 ± 2
10 glyphosate molecules added in a non-vacancy crystal	3.1 ± 0.5
100 glyphosate molecules added in a non-vacancy crystal	7.0 ± 0.5
0 glyphosate molecules added in a 10 vacancy crystal	1.0 ± 0.5
5 glyphosate molecules added in a 10 vacancy crystal	7.6 ± 0.5
10 glyphosate molecules added in a 10 vacancy crystal	2.1 ± 0.8
100 glyphosate molecules added in a 10 vacancy crystal	4.8 ± 0.5

TABLE S1: Solubility results from the different DC simulations expressed as molarity

our forcefield, maintaining the experimentally observed ratio of 25:75 at pH = 1.9 for zwitterionic:charged glycine. We analyzed the different glycine species individually to examine whether their form (zwitterionic or charged) is related to their behavior. The results are shown in Figure S4, where we can observe how both species have maxima, which are located at the interface between glyphosate and the aqueous solution, exhibiting a similar behaviour.

The main difference between them that we observe in Figure S4 is that although zwitterionic glycine is present in smaller proportion, it exhibits a stronger tendency to adhere to the crystal face compared to the charged glycine. To study this observation we calculated the proportion of glycine at the interface and within the bulk. The results of this analysis are presented in Table S2, showing quantitative differences in the percentages and indicating that zwitterionic glycine forms a stronger interfacial coating. One explanation for this phenomena is the possibility of charged glycine of inducing dipoles in water, therefore observing more affinity for water than its zwitterionic counterpart. The density profiles for the different glycine experiments are shown in S5

TABLE S2: Proportion of glycine by location

	% Glycine at the interface	% Glycine in bulk
Charged glycine	33.1	66.9
Zwitterionic glycine	55.5	45.5

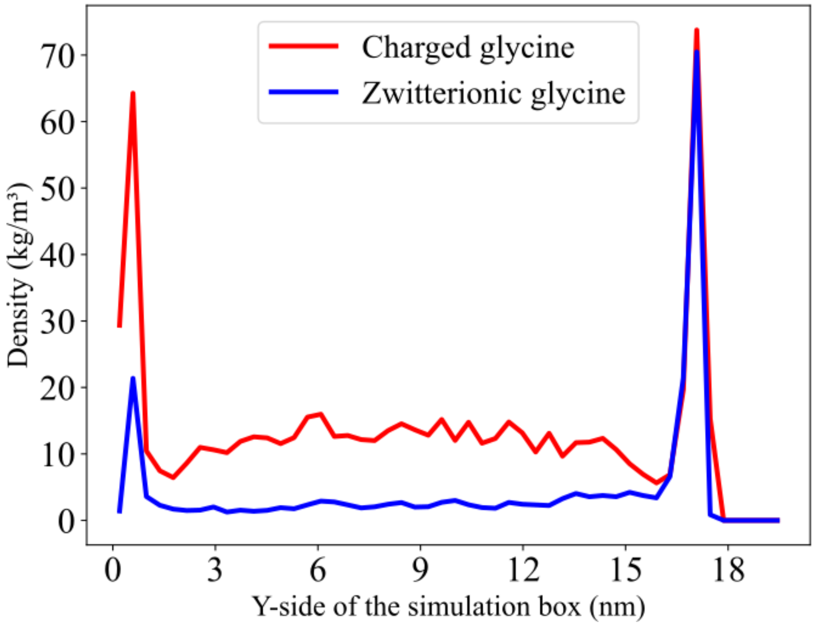

FIG. S4: Density profile of the different glycine species.

The density profiles for the different glycine experiments are shown in S5

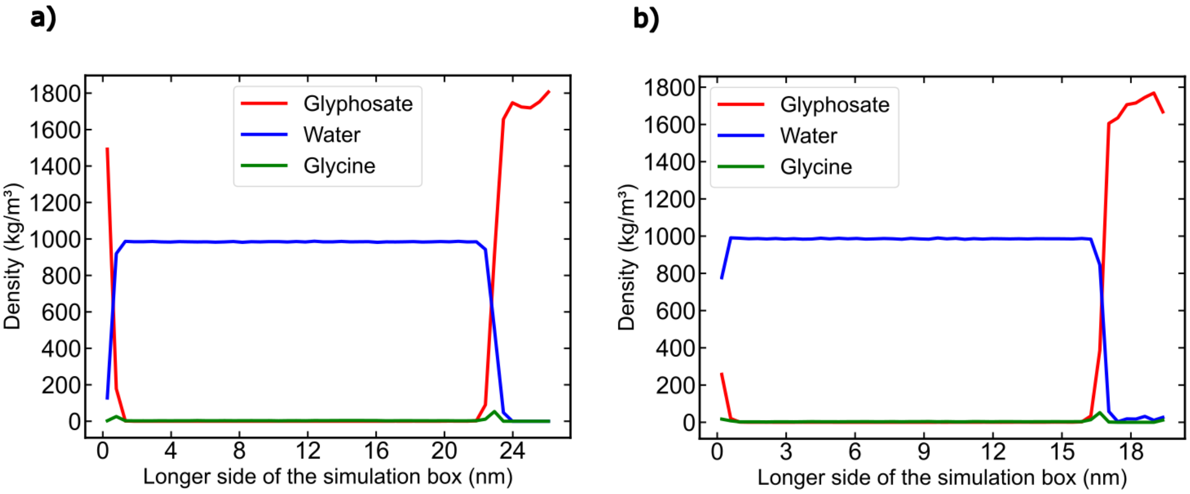

FIG. S5: Glycine density profiles. a) Z-axis simulation with equimolar conditions b) Y-axis simulation with equimolar conditions

SVI. FEP+

The systems studied were comprised of the molecules shown in Table S3. In Figure S6 we show the time evolution of the solvation free energy for the different seeds, which are depicted with different colours.

TABLE S3: Number of molecules in the different systems studied

%wt glycine	H_2O	glyphosate molecules	charged glycine	zwitterion glycine	total glycine
0	15000	1	0	0	0
0.5	15000	1	14	4	18
1	15000	1	27	9	36
2	15000	1	55	18	73

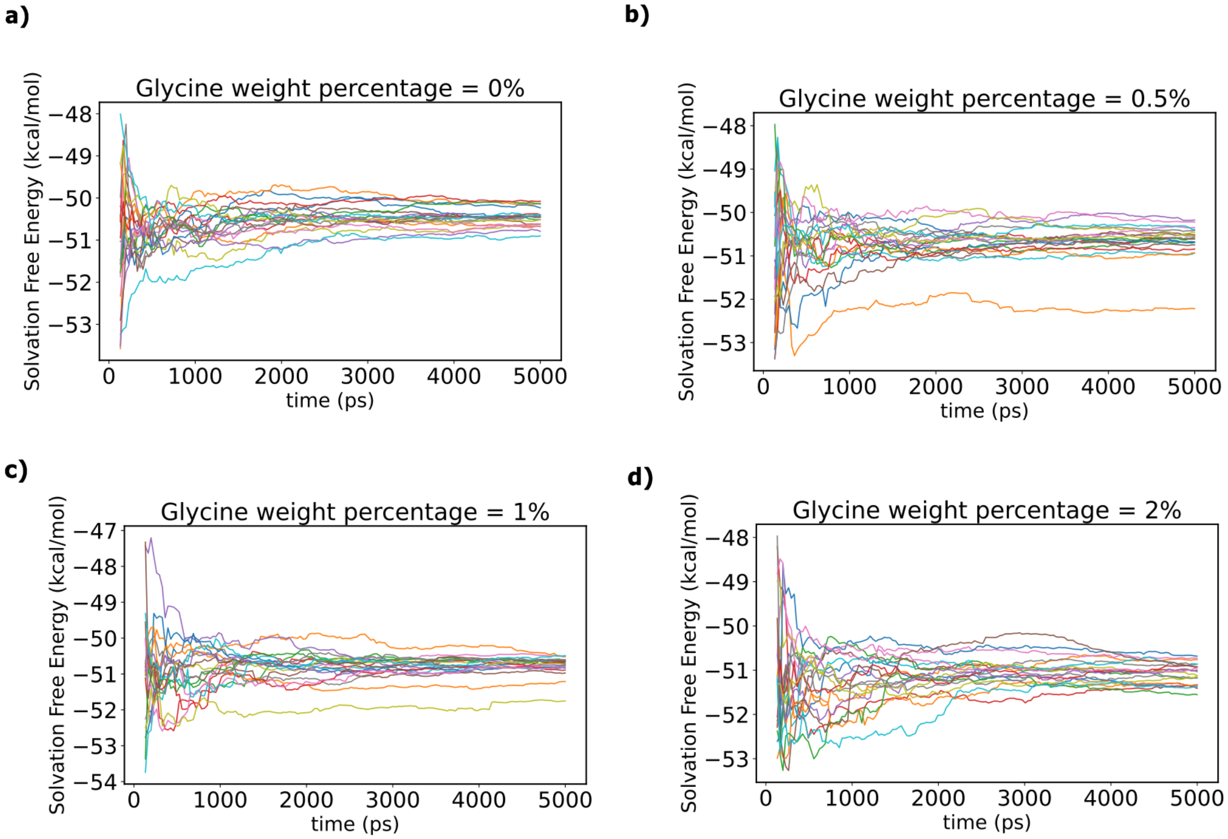

FIG. S6: Solvation free energy *vs* time for each of the 20-seeds (depicted with different colours) for each glycine concentration used.

SVII. ERROR CALCULATION

In this section we will discuss how errors were obtained. For the individual simulations shown in S1, we used the propagation of errors across S8, obtaining the expression shown in S9

$$\Delta m_{\text{glyphosate}} = m_{\text{glyphosate}} \cdot \left[\frac{\Delta \rho_y^{\text{glyphosate}}}{\rho_y^{\text{glyphosate}}} + \frac{\Delta \rho_y^{\text{H}_2\text{O}}}{\rho_y^{\text{H}_2\text{O}}} \right], \tag{S9}$$

For final solubility results we used the standard statistical error obtained with S10.

$$\sigma = \sqrt{\frac{1}{N} \sum_{i=1}^{N} (x_i - \mu)^2}, \tag{S10}$$

where x_i is the i-th observation in the dataset, N is total number of observations in the population, μ is the population mean and σ is population standard deviation

For the FEP+ error, it is first necessary to define how the BAR method calculates the variance. The variance is determined from the curvature of the log-likelihood at the optimum, as shown in Eq. S11.

$$\sigma^2(\Delta G) \approx \left[-\frac{\partial^2}{\partial(\Delta G)^2} \ln L(\Delta G) \Big|_{\Delta G = \Delta G^*} \right]^{-1}, \tag{S11}$$

where ΔG^* is the optimal solution to the BAR equation and L is the likelihood function for the free energy difference ΔG, defined in S12

$$L(\Delta G) = \prod_{i=1}^{N_A} f\Big(-\beta(\Delta U_i^{A \to B} - \Delta G)\Big) \prod_{j=1}^{N_B} f\Big(\beta(\Delta U_j^{B \to A} - \Delta G)\Big), \tag{S12}$$

where $f(x) = \frac{1}{1+e^x}$ is the Fermi function, $\beta = 1/k_B T$, and N_A, N_B are the number of samples from states A and B respectively.

Taking the logarithm in S12, the log-likelihood is:

$$\ln L(\Delta G) = \sum_{i=1}^{N_A} \ln f\Big(-\beta(\Delta U_i^{A \to B} - \Delta G)\Big) + \sum_{j=1}^{N_B} \ln f\Big(\beta(\Delta U_j^{B \to A} - \Delta G)\Big). \tag{S13}$$

The BAR free energy estimate ΔG^* is obtained by maximizing the log-likelihood. Equation S11 provides the standard error of the free energy difference computed by BAR.

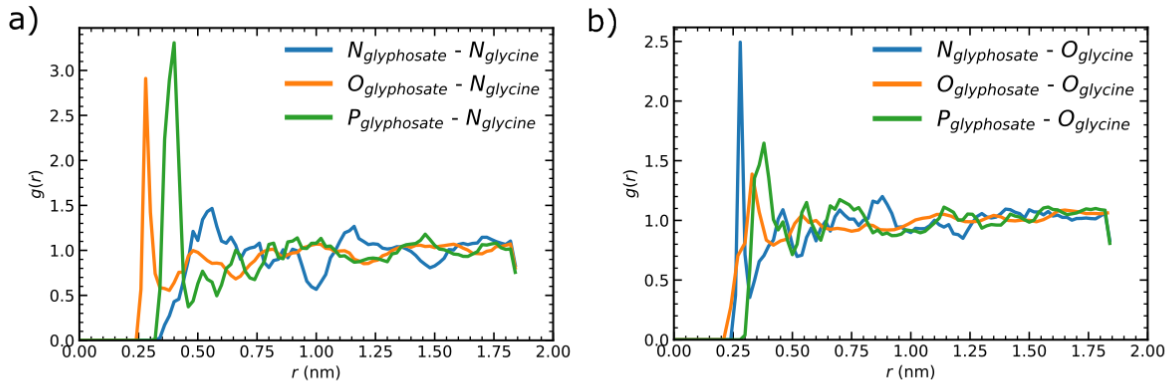

FIG. S7: a) Radial distribution functions g(r) between the N, O, and P atoms of glyphosate and the N atom of glycine as a function of distance r. b) Radial distribution functions, g(r), between the N, O, and P atoms of glyphosate and the O atom of glycine as a function of distance r.

SVIII. RADIAL DSITRIBUTION FUNCTIONS

In order to characterize which are the most relevant glycine-glyphosate interactions driving the increase in solubility, we compute the radial distribution function (g(r)) between the different heavy atoms of both molecules. The results are summarized in Figure S7. From the radial distribution functions between glycine's nitrogen with gluphosate heavy atoms, we conclude that the most remarkable interaction is that between glyphosate's oxygen (negative dipole moment of the molecule) with glycine's nitrogen (positive dipole moment of the molecule), which is expected given the high amount of oxygen atoms (5 in every molecule) per glyphosate, all of which have a negative dipole moment. The following peaks ($P_{Glyphosate}$-$O_{Glycine}$ and $N_{Glyphosate}$-$O_{Glycine}$) appear naturally due to the proximity of these atoms in the neighbouring molecules, binded by oppositely charged groups. From the radial distribution functions between glycine's oxygen and glyphosate heavy atoms, we do not clearly observe a first coordination shell between any pairs of atoms, highlighting the stabilizing role of the $O_{Glyphosate}$-$N_{Glycine}$ pair.

SIX. MOLECULAR DYNAMICS SYSTEM SIZE

In tables S4 and S5 we explicitely state the number of molecules of each species for each simulation mentioned in the main text.

TABLE S4: Number of molecules for glyphosate simulations.

	**Crystal simulations**		**Bulk concentrations**			
**Component**	No vacancies	Vacancies	0 m	0.034 m	0.069 m	0.69 m
Water molecules	–	–	8000	8000	8000	8000
Glyphosate molecules	256	246	0	5	10	100

TABLE S5: Number of molecules for glycine simulations.

	**Crystal simulation**	**Bulk concentrations**	
**Component**	No vacancies	0.5 %	2 %
Water molecules	–	8000	8000
Glyphosate molecules	256	10	10
Glycine molecules	–	10	40

[1] O. F. F. Initiative, "The open force field initiative: Open software and open science," *The Journal of Physical Chemistry B*, vol. 123, no. 17, pp. 3651–3661, 2019. Accessed: 2025-09-08.

[2] Y. Qiu, C. D. Stern, D. G. A. Smith, S. Boothroyd, J. R. Wagner, J. T. Horton, A. Rizzi, T. Gokey, V. T. Lim, J. A. Mitchell, D. L. Mobley, J. D. Chodera, C. I. Bayly, M. R. Shirts, L.-P. Wang, and O. F. F. Consortium, "Open force field sage 2.0.0: Evaluating improvements in energetics and optimized geometries," *Journal of Chemical Theory and Computation*, vol. 19, no. 2, pp. 615–636, 2023.

[3] C. C. Bannan, C. I. Bayly, J. D. Chodera, and D. L. Mobley, "An open source tool for automatically generating smirks patterns," *ChemRxiv*, 2019. Accessed: 2025-09-08.

[4] M. Abraham, A. Alekseenko, C. Bergh, C. Blau, E. Briand, M. Doijade, S. Fleischmann, V. Gapsys, G. Garg, S. Gorelov, G. Gouaillardet, A. Gray, M. E. Irrgang, F. Jalalypour, J. Jordan, C. Junghans, P. Kanduri, S. Keller, C. Kutzner, J. A. Lemkul, M. Lundborg, P. Merz, V. Miletić, D. Morozov, S. Páll, R. Schulz, M. Shirts, A. Shvetsov, B. Soproni, D. van der Spoel, P. Turner, C. Uphoff, A. Villa, S. Wingbermühle, A. Zhmurov, P. Bauer, B. Hess, and E. Lindahl, "Gromacs 2023.2 manual," July 2023.

[5] C. Lu, C. Wu, D. Ghoreishi, W. Chen, L. Wang, W. Damm, G. A. Ross, M. K. Dahlgren, E. Russell, C. D. Von Bargen, R. Abel, R. A. Friesner, and E. D. Harder, "Opls4: Improving force field accuracy on challenging regimes of chemical space," *Journal of Chemical Theory and Computation*, vol. 17, no. 7, pp. 4291–4300, 2021.

[6] C. Vega, E. Sanz, J. Abascal, and E. Noya, "Determination of phase diagrams via computer simulation: methodology and applicationsto water, electrolytes and proteins," *Journal of Physics: Condensed Matter*, vol. 20, no. 15, p. 153101, 2008.

[7] Schrödinger, LLC, *Schrödinger Release 2023-1: Maestro*. Schrödinger, New York, NY, 2023. Release 2023-1.

[8] S. Nosé, "A unified formulation of the constant temperature molecular dynamics methods," *The Journal of Chemical Physics*, vol. 81, no. 1, pp. 511–519, 1984.

[9] G. J. Martyna, D. J. Tobias, and M. L. Klein, "Constant pressure molecular dynamics algorithms," *The Journal of Chemical Physics*, vol. 101, no. 5, pp. 4177–4189, 1994.

[10] M. Tuckerman, B. J. Berne, and G. J. Martyna, "Reversible multiple time scale molecular dynamics," *The Journal of Chemical Physics*, vol. 97, no. 3, pp. 1990–2001, 1992.

[11] M. Parrinello and A. Rahman, "Crystal structure and pair potentials: A molecular-dynamics study," *Physical Review Letters*, vol. 45, no. 14, pp. 1196–1199, 1980.

[12] G. Bussi, D. Donadio, and M. Parrinello, "Canonical sampling through velocity rescaling," *The Journal of Chemical Physics*, vol. 126, no. 1, p. 014101, 2007.

[13] R. W. Hockney, S. Goel, and J. Eastwood, "Quiet high-resolution computer models of a plasma," *Journal of Computational Physics*, vol. 14, no. 2, pp. 148–158, 1974.

[14] L. Yu and K. Ng, "Glycine crystallization during spray drying: the ph effect on salt and polymorphic forms," *Journal of pharmaceutical sciences*, vol. 91, no. 11, pp. 2367–2375, 2002.
